# Comparing the durability of the long-lasting insecticidal nets DawaPlus^®^ 2.0 and DuraNet© in northwest Democratic Republic of Congo

**DOI:** 10.1186/s12936-020-03262-0

**Published:** 2020-05-24

**Authors:** Paul Mansiangi, Solange Umesumbu, Irène Etewa, Jacques Zandibeni, Nissi Bafwa, Sean Blaufuss, Bolanle Olapeju, Ferdinand Ntoya, Aboubacar Sadou, Seth Irish, Eric Mukomena, Lydie Kalindula, Francis Watsenga, Martin Akogbeto, Stella Babalola, Hannah Koenker, Albert Kilian

**Affiliations:** 1 Ecole de Santé Publique, Université de Kinshasa, Kinshasa, Democratic Republic of Congo; 2 National Malaria Control Programme, Kinshasa, Democratic Republic of Congo; 3 PMI VectorWorks Project, JHU Center for Communication Programs, Baltimore, MD USA; 4 U.S. President’s Malaria Initiative, U.S. Agency for International Development, Kinshasa, Democratic Republic of Congo; 5 U.S. President’s Malaria Initiative, Centers for Disease Control and Prevention, Atlanta, GA USA; 6 Institut Nationale de Recherche Bio-Medicale, Kinshasa, Democratic Republic of Congo; 7 Centre de Recherche Entomologique de Cotonou, Cotonou, Benin; 8 PMI VectorWorks Project, Tropical Health LLP, Montagut, Spain

**Keywords:** LLIN durability, Monitoring, DRC

## Abstract

**Background:**

Anecdotal reports from DRC suggest that long-lasting insecticidal nets (LLIN) distributed through mass campaigns in DRC may not last the expected average three years. To provide the National Malaria Control Programme with evidence on physical and insecticidal durability of nets distributed during the 2016 mass campaign, two brands of LLIN, DawaPlus^®^ 2.0 and DuraNet**©**, were monitored in neighbouring and similar health zones in Sud Ubangi and Mongala Provinces.

**Methods:**

This was a prospective cohort study of representative samples of households from two health zones recruited at baseline, 2 months after the mass campaign. All campaign nets in these households were labelled, and followed up over a period of 31 months. Primary outcome was the “proportion of nets surviving in serviceable condition” based on attrition and integrity measures and the median survival in years. The outcome for insecticidal durability was determined by bio-assay from subsamples of campaign nets.

**Results:**

A total of 754 campaign nets (109% of target) from 240 households were included in the study. Definite outcomes could be determined for 67% of the cohort nets in Sud Ubangi and 74% in Mongala. After 31 months all-cause attrition was 57% in Sud Ubangi and 76% in Mongala (p = 0.005) and attrition due to wear and tear was 26% in Sud Ubangi and 48% in Mongala (p = 0.0009). Survival in serviceable condition at the last survey was 37% in Sud Ubangi and 17% in Mongala (p = 0.003). Estimated median survival was 1.6 years for the DawaPlus^®^ 2.0 in Mongala (95% CI 1.3–1.9) and 2.2 years for the DuraNet in Sud Ubangi (95% CI 2.0–2.4). Multivariable Cox proportionate hazard models suggest that the difference between sites was mainly attributable to the LLIN brand. Insecticidal effectiveness was optimal for DuraNet**©**, but significantly dropped after 24 months for DawaPlus^®^ 2.0.

**Conclusions:**

In the environment of northwest DRC the polyethylene LLIN DuraNet**©** performed significantly better than the polyester LLIN DawaPlus^®^ 2.0, but both were below a three-year median survival. Improvement of net care behaviours should be able to improve physical durability.

## Background

Based on its location around the equator and its ecology, the Democratic Republic of Congo (DRC) belongs to the countries with very high malaria transmission potential in most of its territory. It was estimated that in 2017 DRC contributed 11% of all malaria cases world-wide, second only to Nigeria and hence DRC is one of the focus countries of the World Health Organization’s high burden, high impact initiative [1]. The primary intervention of the National Malaria Control Programme (NMCP) for vector control is the distribution of insecticide treated nets (ITN) which today are almost exclusively long-lasting insecticidal nets (LLIN). These have been shown to significantly reduce malaria parasite prevalence in DRC by up to 44%, especially if high community-level coverage and use is achieved [2]. A recent geospatial analysis of all-cause child mortality based on data from two DRC Demographic and Health Surveys (2007 and 2014/15) also demonstrated a 41% mortality reduction associated with ITN distributions [3]. The NMCP uses a strategy of “rolling” LLIN mass campaigns to repeatedly cover all provinces where implementation is stretched over longer periods moving from one province to another. There is evidence that these campaigns can reach the “universal coverage” target of 80% population access to ITNs [4] even though distributions can be challenging in some settings such as those with internally displaced persons [5].

The general World Health Organization (WHO) recommendation on the frequency of LLIN mass distribution campaigns is every 3 years based on the assumption that the median survival of the products is 3 years [6]. The guidance document, however, also highlights that the longevity of LLIN can vary significantly between communities or settings and recommends that countries monitor the physical and insecticidal durability of the LLIN brands they distribute following the most recent methodological guidance [7–9].

In the DRC, some anecdotal reports from the field have suggested that the average survival of LLIN under operational conditions may be less than 3 years. To date, only one LLIN durability study has been conducted in DRC. The study was carried out in 2015 with a retrospective design for the survival aspects of ITN and a cross-sectional one for aspects related to the use of LLINs in households [10]. The results suggest that median survival in serviceable condition of the campaign nets assessed (mainly PermaNet^®^ 2.0) was generally lower than expected and varied in the eight provinces covered between 1.3 and 3.1 years. Collecting further evidence on the durability of LLIN, therefore, is a priority of the NMCP to inform its decisions on LLIN distribution strategies.

The objectives of the present study were to (i) compare physical and insecticidal durability of two LLIN brands, DawaPlus^®^ 2.0 and DuraNet**©**, distributed during the 2015/16 mass campaign in two similar health zones in northwest DRC using a prospective cohort design, and (ii) identify major determinants influencing LLIN durability.

## Methods

### Study sites

Two neighbouring provinces in northwest DRC, Sud Ubangi and Mongala, were selected as they had received different brands of LLIN during the most recent campaign. One health zone (HZ) along the provincial border was purposively selected in each province: Ndage HZ for Sud Ubangi and Binga HZ for Mongala.

**Fig. 1:**
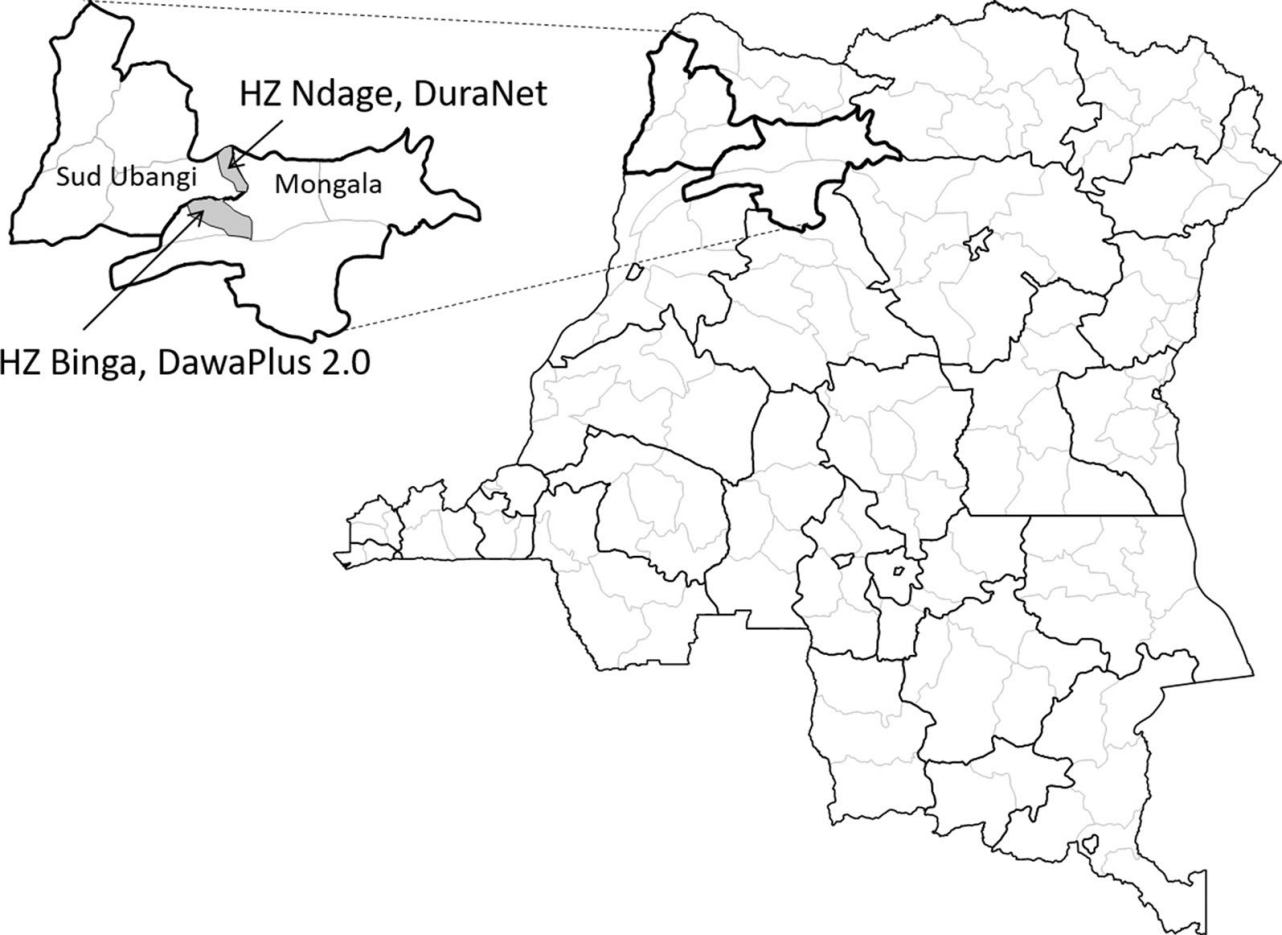
Location of study sites within DRC

The locations are shown in Fig. 1 and can be described briefly as follows:

The climate is equatorial (warm and humid), with a bimodal rain pattern: the rainy season usually last 9 months starting from March to November. The dry season starts from December to March, and 3 weeks in the month of July. The vegetation is dominated by the equatorial forest with clay-sandy soil. The hydrography of the area is composed of three large rivers: the Congo River, the Mongala River, and the Sambo River. The tributaries of these rivers intersect the HZs thus making access difficult for some places, especially during the rainy season.

In general, agriculture, hunting, fishing and small trade are the main occupations of the population. Livestock production is common and mainly concerns small livestock and poultry. In addition, there are a number of large agricultural companies active such as the Société des Cultures au Congo which is the main employer in the Binga HZ. It specializes in the production of palm oil, rubber and cocoa. It employs about 30% of the labour force available in this HZ. It is thanks to the presence of this company that the HZ has telecommunication coverage at least in some areas.

Malaria is the most dominant disease in terms of morbidity and mortality with perennial transmission and hyper- to holoendemic endemicity. Based on estimates from the Malaria Atlas Project (https://map.ox.ac.uk), *Plasmodium falciparum* prevalence among children 2–10 years is around 60%. Other diseases with high morbidity are waterborne diseases, acute respiratory infections, and protein-energy malnutrition.

### Study design

This was a prospective study of representative cohorts of LLINs distributed during the 2015/16 mass distribution campaigns and followed for up to 3 years. The design was based on the guidance from the U.S. President’s Malaria Initiative for LLIN durability monitoring [11] and in this case comparing the durability of the two different LLIN brands between the two very similar locations. The first brand was DawaPlus^®^ 2.0, a 100-denier polyester LLIN in white colour and distributed in Mongala Province. This LLIN brand uses the coating technology with a loading dose of 80 mg/sq m of deltamethrin and obtained interim WHOPES recommendation in July 2009 [12] and WHO prequalification in January 2018 [13]. In March 2019, the prequalification was transferred to a new manufacturer (NRS Moon Netting FZE) and the product is now called Tsara Soft^®^ listed under prequalification number 028-003. The second LLIN brand was DuraNet©, a 150-denier polyethylene LLIN in blue colour which uses incorporation technology with a loading dose of 260 mg/sq m of alphacypermethrin. DuraNet© received full WHOPES recommendation in July 2013 [14] and WHO pre-qualification status in December 2017 [13].

Within 6 months of the respective mass distribution campaigns LLINs were to be sampled and followed up after 12, 24, and 36 months through household surveys. At each time point measures of physical durability were assessed (attrition and integrity) using a household questionnaire and net damage assessment tools. For all data points after baseline, 30 campaign nets per site were sampled and retrieved for assessment of insecticidal effectiveness (bio-assay).

### Sample size and sampling

Sample size was calculated to be sufficient to find a difference of ± 9%-points from a 50% LLIN survival point estimate after 3 years as significant at the 95% confidence level or an 18% difference between the two LLIN brands. Further assumptions were a power of 80%, design effect of 2.5, all-cause attrition of 35% and attrition due to wear and tear of 20% over 3 years [15], an initial household non-response rate of 5%, campaign distribution of one LLIN for every two people with rounding up for odd-numbered households, and an initial loss between campaign and baseline survey of 8% of the campaign nets. This is equivalent to a deviation from the assumed 3-year median survival by 10–12 months and resulted in the need for a cohort of 345 campaign nets to be recruited per site. Based on an estimated average household size of five persons this required 150 households sampled from 15 clusters with 10 households each.

Clusters (communities) were sampled with probability proportionate to size using the campaign registration lists as sampling frame after inaccessible communities had been eliminated from the sampling frame. Households within clusters were selected using simple random sampling from lists of eligible households prepared by the field teams on the day of the survey. For communities with more than 200 households a segmentation approach was used and only the selected segment was sampled. Up to five replacement households were sampled per cluster to substitute in case a sampled household had not received nets from the campaign or did not consent to participate. Within each household all LLINs identified as from the campaign by brand, colour and report by the respondent were labelled with a unique ID number and bar-code for future follow-up, even when they were still in the package at the time of the baseline survey.

Campaign nets for bio-assay testing were sampled from the cohort only at the final survey using simple random sampling. For the 12- and 24-months surveys campaign nets were sampled from neighbouring households as follows: within each cluster two or three index households were randomly identified from the cohort and when the field teams reached these households, they went left to the next neighbour that had campaign nets and consented to give them up for the study. A brief questionnaire was filled for these nets regarding use and washing. For all LLINs collected for bio-assay new replacement LLINs were given.

### Field procedures

An implementation team of nine individuals was established per site, with one overall site coordinator and two field teams each consisting of one supervisor and three interviewers. Activities in the field were overseen by staff of the Kinshasa School of Public Health in cooperation with NMCP staff. Interviewers and supervisors were carefully selected so that they were culturally acceptable, had good knowledge of the local languages and experience in conducting household surveys.

A 5-day training was held at baseline and 3-day refresher trainings before each follow-up survey. Special emphasis was put on the process of a standardized assessment of net damage using a template to identify hole size categories and tallying hole counts using an application on the digital devices used for data entry. The questionnaire had three main modules: one for the household respondent, a second for the cohort campaign nets (including nets lost between campaign and baseline survey), and a third module for other nets owned by the household at each time point. In addition, a list of household members and assets was obtained at baseline and at the final survey. GPS coordinates were recorded at baseline and used to track household during follow up. If households moved within the clusters the new homes were identified, if they moved outside the cluster, they were considered lost to follow-up.

The mass distribution campaigns took place 12–16 August 2016 in Sud Ubangi and 25–28 August 2016 for Mongala. Baseline assessments took place October 2016, the 12 months data collection was carried out August 2017, the 24-months surveys were done in May 2018, and the final survey took place March 2019. The earlier dates for the last two surveys were chosen to avoid the heavy rains and in view of the pending close-out of the PMI VectorWorks project. The questionnaire and all other tools are publicly available (http://www.durabilitymonitoring.org).

### Laboratory analysis

Bio-assays were done at the Kinshasa National Institute for Biomedical Research while those for the 36-months samples were done at the Centre for Entomological Research in Cotonou, Benin using the standard WHO bio-assay cone test procedure [8]. A pyrethroid-susceptible strain of *Anopheles gambiae* sensu lato was used with five mosquitoes per cone, five sites tested on each net (four sides and roof panel) and two cones per location. This procedure was repeated twice for a total of 20 cone tests with 100 mosquitoes per net. Recorded were 60-min knock-down (KD60) and 24-h functional mortality and then combined as optimal insecticidal effectiveness (KD60 ≥ 95% or functional mortality ≥ 80%), minimal effectiveness (KD60 ≥ 75% or functional mortality ≥ 50%), or failure (not reaching minimal effectiveness criteria) [7].

### Data management

For data collection, tablet PCs were used and installed with the Open Data Kit (ODK) software for the questionnaire and Open Street Map for Android (OSMAND) for household tracking. Data from each field team was collected daily on a local storage device by the coordinator until it could be transferred to a central data base. Data was converted from ODK to comma-delimited data files using the ODK briefcase tool for inspection of incoming data and feedback was provided to the teams. For each survey round updated lists were compiled from the household and cohort net master files and preloaded on the ODK system including all households and cohort nets for which no definite outcome was available to date. After completion of the surveys, datasets were transferred to Stata version 14.2 (Stata, Texas, USA) for further aggregation, consistency checks and preparation for analysis. Stata do-files (macros) developed by the PMI VectorWorks project were applied and adjusted as needed [11]. For the final analysis data sets from all four surveys were merged and a duration format data set prepared for survival analysis.

### Data analysis

#### Definition of outcomes

The primary outcome measure was the physical net survival and was defined as the proportion of cohort nets received from the LLIN campaign still in serviceable physical condition (definition provided below) [7]. For the calculation of this outcome two interim outcomes were calculated as follows:

*Net attrition rate due to wear and tear* was defined as the proportion of originally received nets which were lost due to wear and tear (thrown away, destroyed or used for other purposes) at the time of assessment. Nets received but given away for use by others or stolen were excluded from the denominator. Similarly, nets with unknown outcome were excluded.

*Net integrity* was measured first by the proportionate Hole Index (pHI) as recommended by WHO [8]. Holes in cohort LLINs were counted categorized into four different sizes: size 1: 0.5–2 cm, size 2: 2–10 cm, size 3: 10–25 cm and size 4: larger than 25 cm in maximum diameter. The proportionate Hole Index (pHI) for each net was then calculated as the number of holes counted multiplied by the size category weights as suggested by the WHO [8], namely 1, 23, 196, and 576 for the four hole sizes respectively. Based on the pHI each net was then categorized as “good”, “damaged”, “serviceable” or “torn” as follows [8]:

Good: total hole surface area < 0.01 sq m or pHI < 64

Damaged: total hole surface area 0.01–0.1 sq m or pHI 65–642

Torn: total hole surface area > 0.1 sq m or pHI > 642

Serviceable: total hole surface area ≤ 0.1 sq m or pHI ≤ 642 (good or damaged)

In order to be able to compare physical survival measured at different time points the outcome of median net survival was estimated defined as the time in years until 50% of the originally distributed LLIN were no longer serviceable. Two approaches were used to estimate median survival. At each time point the proportion surviving in serviceable condition were plotted against the hypothetical survival curves with defined median survival [7] (Additional file 1) and the median survival was taken as the relative position of the data point on a horizontal line between the two adjacent median survival curves. After the final survey median net survival was calculated from at the last two time points provided both were below 85% (when the hypothetical curves are linear), using the following formula:

$${\text{tm}} = {\text{t}}1 + \frac{{\left( {{\text{t}}2 - {\text{t}}1} \right) *\left( {{\text{p}}1 - 50} \right)}}{{\left( {{\text{p}}1 - {\text{p}}2} \right)}}$$

where tm is the median survival time, t1 and t2 the first and second time points in years and p1 and p2 the proportion surviving to first and second time point respectively in percent. Confidence intervals for this estimate were calculated by projecting the 95% CI from the survival estimates in the same way as described above.

### Explanatory variable preparation

Overall household attitudes towards net care and repair were measured using a set of Likert score questions where a statement was read to the respondent (head of household or spouse) and the level of agreement recorded. These were analysed by recoding the four-level Likert scale score to have a value of − 2 for “strongly disagree”, − 1 for “disagree”, +1 for “agree” and +2 for “strongly agree.” These attitude scores for each respondent were then summed and divided by the number of statements to calculate an average household attitude score for which 0 represents a neutral result and positive values a positive result. For each site the proportion of households with a score above 1 (very positive attitude) were calculated at each survey.

Further aggregation of results was done across all four surveys. For household and net risk factors for durability the following categories were used: “never” = responded with “never” in all surveys the household participated; “at times” = household reported the behaviour as “sometimes” in at least one survey round or had conflicting statements; “always” = responded with “always” in all surveys the household participated. Exposure and attitude were similarly aggregated, i.e. “once” = reported exposure or positive attitude score at one of the four survey rounds; “twice or more” = at two or more survey rounds.

A wealth index was calculated for the baseline data set using the basic household assets and a principal component analysis with the first component used as the index. Households were then grouped into tertiles. The full household data collection and wealth index was repeated at the final survey. However, at the 12 and 24 months no specific household or member data were collected.

### Statistical analysis

For continuous variables, arithmetic means were used to describe the central tendency and the *t* test for comparison of groups for normally distributed data. Otherwise, median and Kruskal–Wallis test were used. Proportions were compared by contingency tables and the Chi squared test used to test for differences in proportions. For calculation of confidence intervals around estimates as well as survival analysis models, the intra- and between-cluster correlation has been taken into account.

Data was set up for survival analysis as a duration format data set where each time interval for a net is a separate observation. Analysis was done using an intention to treat approach, i.e. risk of failure was considered to start at the day of distribution irrespective of whether or when the net was hung and used. Failure was defined as a net being lost to wear and tear or torn based on physical assessment (pHI). The time of failure was directly calculated from the reported time of loss by the respondent or taken as the mid-point between the last two surveys if unknown. A secondary analysis used a per-protocol approach where the risk of damage was considered to begin only when a net was first hung. Basic survival analysis was done using Kaplan–Meier estimations of survival function. Determinants of survival were explored using Cox proportionate hazard models. Separate models were constructed for household factors and for net level factors, such that models with net-level factors included only nets that had been ever hung for use during the study. Factors were tested first in individual models which were then used to construct the final multivariable models. Variables compiled across all surveys were left in the final model if they had a p-value of 0.1 or lower. Final model fit was tested using a linktest and Schoenfeld residuals and log–log plots were used to check the proportionate hazard assumption.

## Results

### Sample characteristics

In each of the sites a total of 377 campaign nets were recruited and labelled, 109% of the target. However, since the average household size was higher than anticipated, 7.3 persons in Sud Ubangi and 6.2 in Mongala, the target sample size for cohort nets was already exceeded after 12 of the anticipated 15 clusters. Since no more bar-coded labels were available, the remaining 3 cluster were dropped reducing the recruited households to 120 at each site.

After the final survey round 23% of the households in Sud Ubangi and 41% in Mongala had terminated the study because all the labelled cohort nets had been lost (Table 1). Moving away was the second most common reason for loss to follow up. Only three households refused, all in Sud Ubangi and for 3% of households in Sud Ubangi and 4% in Mongala no follow-up visits could be done after recruitment due to absence. As shown in Table 1, a definite outcome could be established for 67% of the cohort nets in Sud Ubangi and for 74% in Mongala (p = 0.29). While in Sud Ubangi the most common reason for no definite outcome was the household moving away (17%), it was inability to establish the whereabouts of the net from the respondent (14%) in Mongala.

**Table 1:** Follow-up status of recruited households and campaign cohort nets after final survey (31 months)

Variable	Sud Ubangi (DuraNet©)	Mongala (DawaPlus^®^ 2.0)
Households	N = 120	N = 120
Still has any campaign nets	55.0%	45.8%
Lost all their campaign nets	23.3%	40.8%
Moved away	16.7%	9.2%
Refused	2.5%	0.0%
Nobody home at survey	2.5%	4.2%

Campaign cohort nets	N = 377	N = 377
Known outcome	67.4%	74.3%
Unknown outcome	32.6%	25.7%
Household moved away or refused	16.5%	7.4%
Net used elsewhere	7.7%	4.0%
Fate of net unknown	8.5%	14.3%

As was anticipated, the demographic and socio-economic characteristics of the sampled households in the two sites were overall similar but also showed differences (details are shown in Additional file 2). Both areas can be characterized as poor, rural, agricultural communities. House construction was simple with 18% of houses having improved roof materials (sheets), 12% improved walls (plastered) and 1% in Sud Ubangi and 13% in Mongala (p = 0.003) improved floor materials (cement). Cooking fuel was firewood for 96% of households and charcoal for 4%. Access to simple latrines was ubiquitous (99%) but none of the households in Sud Ubangi and only 19% in Mongala had access to safe drinking water (p = 0.007) as most took their water from rivers or creeks. Land for farming was owned by 68% of households, but ownership of livestock differed between the sites with 92% of households in Sud Ubangi and 80% in Mongala (p = 0.015) having livestock, mainly chicken and a few goats. Household assets were very limited, 46% owned a bicycle, 13% a motorbike, none a car. Radios were owned by 48% of households and television by 0.6% in Sud Ubangi and 7% in Mongala (p = 0.007). Ownership of mobile phones was very low and lower in Sud Ubangi (8%) than Mongala (22%, p = 0.03) due to the slightly better network coverage there. Only 10% of households were headed by females and average age of heads of households was 44 years. Educational level of heads of households was high with 64% having had at least some secondary school education and only 22% being non-literate. There was no evidence that the demographic or socio-economic situation had changed during the 3 years of the study.

### Risk factors of physical durability

A number of behavioural factors that are known or thought to be associated with the risk of damage of nets were monitored. These can be divided into four groups: factors of the net use environment in the household, knowledge and attitudes towards net care and repair of the household respondent, net handling and washing, and type of sleeping place. Household related factors depended exclusively on the respondents who were in 58% the head of household, in 32% the spouse and in 10% other adults in the household with no differences between sites.

Seeing rodents or their traces around the house was reported by 86% of households in Sud Ubangi and 95% in Mongala (p = 0.012). Other key variables are shown in Table 2. Storing food in a room used for sleeping is thought to attract rodents which in turn could damage the nets. This practice was similar at the two sites with about three quarter of households doing so at least at times. A difference was seen in cooking in the room were people sleep and hence a net is likely to be hanging. This was more frequent in Sud Ubangi. The two other household level factors monitored were exposure to social and behaviour change communication (SBCC) messages on net use and care in the 6 months preceding each survey and the net care attitude scores based on a set of Likert score questions. Both outcomes were determined across all surveys. At least one message exposure was reported by 74% of households, but exposures at multiple surveys were not very common, reported by only 32% with no difference between sites. Indeed, most of the exposure happened at baseline, 2 months after the campaign when 64% of household in both sites had heard at least one message. This rate than rapidly declined in both sites to 48% after 12 months, 10% after 24 months and 6% after 36 months. Messages in Sud Ubangi were exclusively transmitted through interpersonal communication (IPC), mainly through community health workers (86%), faith-based organizations (40%), and health workers at facilities (36%). In Mongala there was some exposure also through media (17%), mainly radio. IPC was also conducted through community health workers and health facilities, but faith-based organizations did not play a role.

**Table 2:** Net use environment at household level

Variable	Sud Ubangi (DuraNet©)	Mongala (DawaPlus^®^ 2.0)	P-value for site comparison
Households	N = 120 % (95% CI)	N = 120 % (95% CI)	
Storing of food in sleeping rooms			
Never	30.8 (16.6–50.0)	23.3 (13.4–37.4)	0.25
At times	47.5 (35.9–59.4)	63.3 (51.3–73.9)	
Always	21.7 (11.0–38.3)	13.3 (7.8–21.9)	
Cooking in sleeping room			
Never	40.8 (23.8–60.4)	55.8 (39.8–70.8)	0.0001
At times	26.7 (18.7–36.5)	43.3 (28.3–59.7)	
Always	32.5 (16.1–54.6)	0.8 (0.1–6.4)	
Exposure to net use or care messages			
Never	15.0 (8.5–25.2)	17.5 (11.4–26.0)	0.52
Once	55.8 (47.9–63.5)	47.5 (38.1–57.1)	
Twice or more	29.2 (18.3–43.1)	35.0 (24.2–47.5)	
Very positive net care attitude (score > 1.0)			
Never	6.7 (2.5–16.7)	35.8 (26.3–46.7)	0.0002
Once	19.2 (10.8–31.6)	27.5 (20.5–35.8)	
Twice or more	74.2 (56.8–86.3)	36.7 (27.4–47.1)	

Results were aggregated across all four surveys i.e. “never” = responded with “never” in all surveys the household participated; “at times” = household reported the behaviour as “sometimes” in at least one survey round or had conflicting statements; “always” = responded with “always” in all surveys the household participated. Exposure and attitude were similarly aggregated, i.e. “once” = reported exposure or positive attitude score at one of the four survey rounds; “twice or more” = at two or more survey rounds

Durability risk factors regarding the handling and use of nets when hanging are shown in Table 3. Out of the eight criteria considered, five were the same in both sites. Almost none of the cohort nets was ever found folded or tied up when hanging to take it out of harm’s way during the day. About one-third of the nets were used only by adults throughout the follow-up while 12% were exclusively used by children. Around 80% of the cohort nets seen in the surveys were ever reported to have been washed with a median of two washed per 6-month period or four washes per year. When washed, the sometimes milder “country soap” was used exclusively for 72% of cohort nets while 12% used exclusively detergents. The biggest difference found between the sites was the location of drying washed nets. In Sud Ubangi 76% of the washed cohort nets were always dried inside and only 14% always outside. The situation was reversed in Mongala were 82% of washed cohort nets were exclusively dried outside. However, since 85% of these nets were never dried over fences or bushes and only 3% always, the risk of damage through the drying process was only marginally higher in Mongala compared to Sud Ubangi. The other variable for which a difference between the sites was found was the type of sleeping place over which the cohort net was used. At both sites few nets were consistently used over a finished bedframe, but more nets in Mongala were used over foam mattresses with or without a wooden platform.

**Table 3:** Net use environment and washing of cohort nets from campaign

Cohort nets	Sud Ubangi (DuraNet©)	Mongala (DawaPlus^®^ 2.0)	P-value for site comparison
	N = 377 % (95% CI)	N = 377 % (95% CI)	
Ever hung	82.2 (74.5–88.0)	54.6 (45.6–63.4)	0.0001
Ever used	81.7 (73.6–87.7)	54.9 (45.9–63.6)	0.0001

Cohort nets ever hung	N = 310	N = 206	
Tied up or folded when hanging			
Never	97.1 (80.0–99.6)	95.6 (79.2–99.2)	0.73
At times	1.6 (0.2–11.8)	2.9 (0.5–16.3)	
Always	1.3 (0.1–9.6)	1.5 (0.3–6.5)	
Type of sleeping place**			
Bed frame (finished)	0	6.8 (3.9–11,7)	0.012
Bed frame (sticks)	46.8 (22.8–72.3)	33.7 (24.3–44.5)	
Foam mattress	2.9 (0.9–8.6)	22.0 (13.8–33.1)	
Reed mat	50.3 (24.0–76.4)	37.6 (26.5–50.0)	

Cohort nets ever used	N = 296	N = 204	
Dominant user group			
Children only	10.1 (7.3–13.8)	13.7 (8.2–22.1)	0.40
Children with adults	57.1 (50.4–63.5)	51.0 (44.4–57.5)	
Adults only	32.8 (28.5–37.4)	35.3 (26.1–45.8)	
Ever washed	81.8 (75.2–87.0)	76.8 (66.9–84.4)	0.32

Cohort nets ever washed	N = 257	N = 176	
Washes last 6 months	2.0 (1.5–3.0)	2.0 (1.5–3.0)	0.89
Median (IQR)			
Use of detergent			
Never	73.5 (57.2–85.2)	69.3 (58.3–78.5)	0.68
At times	16.3 (9.5–26.6)	17.0 (11.9–23.9)	
Always	10.1 (5.2–18.7)	13.6 (7.4–23.8)	
Drying net outside			
Never	75.5 (64.1–84.2)	6.8 (3.2–13.9)	< 0.0001
At times	10.9 (6.7,17.3)	11.4 (6.1–20.3)	
Always	13.6 (8.7–20.6)	81.8 (71.0–89.2)	
Drying over bush or fence			
Never	95.3 (88.5–98.2)	85.8 (77.3–91.5)	0.034
At times	2.7 (0.9–8.3)	10.8 (5.1–21.5)	
Always	1.9 (0.7–5.2)	3.4 (1.4–8.0)	

** Lowest type of sleeping place ever reported for net

*** Average of all recoded 6 months episodes for each net

**Fig. 2:**
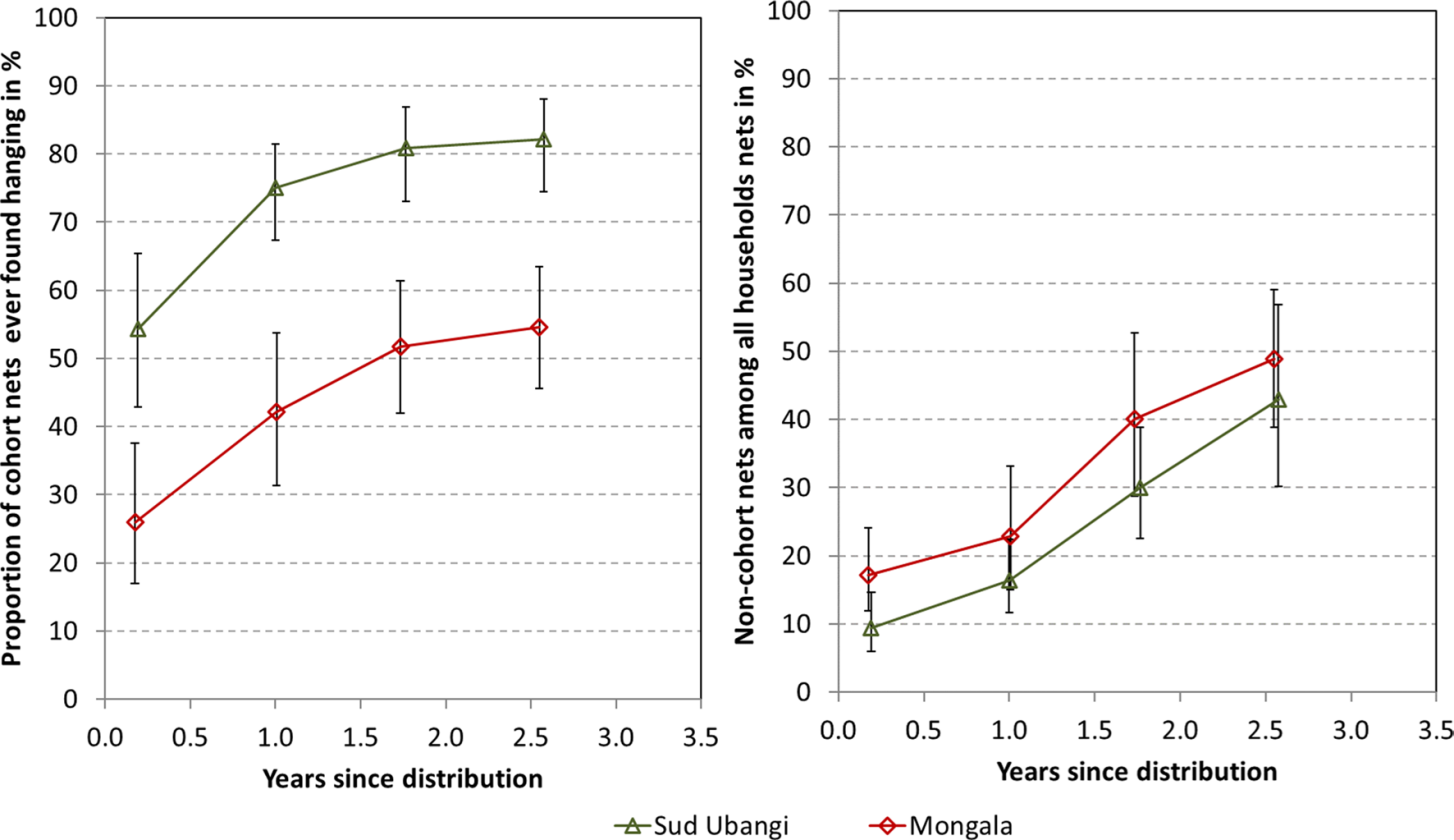
Cohort nets ever found hanging (left) and share of non-cohort nets among household net crop (right)

At baseline, 2 months after the campaign, only 54% of the cohort nets in Sud Ubangi and 26% in Mongala were found hanging while 43% and 71% respectively still were in their packages. The proportion of cohort nets ever found hanging thereafter increased rapidly to reach 81% after 24 months in Sud Ubangi and 52% in Mongala (Fig. 2 left). There was only minimal further increase in ever hanging nets at the final survey (Table 3), but at all times the hanging rate in Sud Ubangi was significantly above that in Mongala. However, at baseline 23% of households in Sud Ubangi and 39% in Mongala also owned other nets not obtained from the recent mass campaign and of these nets 81% at both sites were hanging. Over the follow-up period households owning any non-cohort nets continuously increased in Sud Ubangi reaching 64% at the final survey while it fluctuated around 40% in Mongala. This constant influx of new nets after the campaign resulted in an increasing proportion of the non-cohort nets among all nets owned by the households reaching 43% in Sud Ubangi and 43% in Mongala (Fig. 2 right). At 12 months, a significant part of non-cohort nets was received from friends and family (44% Sud Ubangi and 29% Mongala) and these were most likely campaign nets not needed by other households. Nets from the private sector played a minor role in Sud Ubangi (maximum 14%), although the relative contribution of this source increased over time, and played a more significant role in Mongala where at the end of the study 41% of the non-cohort nets were reported to have been obtained from the private sector. In both sites the majority of nets from the private sector could be identified as LLIN (88% in Sud Ubangi and 75% in Mongala, p = 0.3). Hanging of cohort nets was also found to be a function of the physical condition of the net. If a net was moderately damaged (pHI 65–300) 92% of cohort nets were hanging. This rate decreased to 84% for more damaged nets (pHI 301–642) and to 74% for torn nets (pHI > 642, p = 0.0002). This relationship was constant between sites and surveys. When all nets owned by the households, cohort and non-cohort nets, were considered, 79% of them were hanging at 24 months in Sud Ubangi and 83% at the final survey. For Mongala the hanging rates were 72% and 75%, respectively (p = 0.35 and p = 0.08 for site comparisons).

Use of cohort as well as non-cohort was strongly associated with nets hanging, i.e. 99% of nets that were observed hanging during the surveys were reported to have been used the previous night while only 6% of those not hanging were reported used. There was no evidence of seasonal variation in net use as in both sites > 85% of respondents said they used the nets equally during the rains and the “dry” season.

#### Attrition

**Fig. 3:**
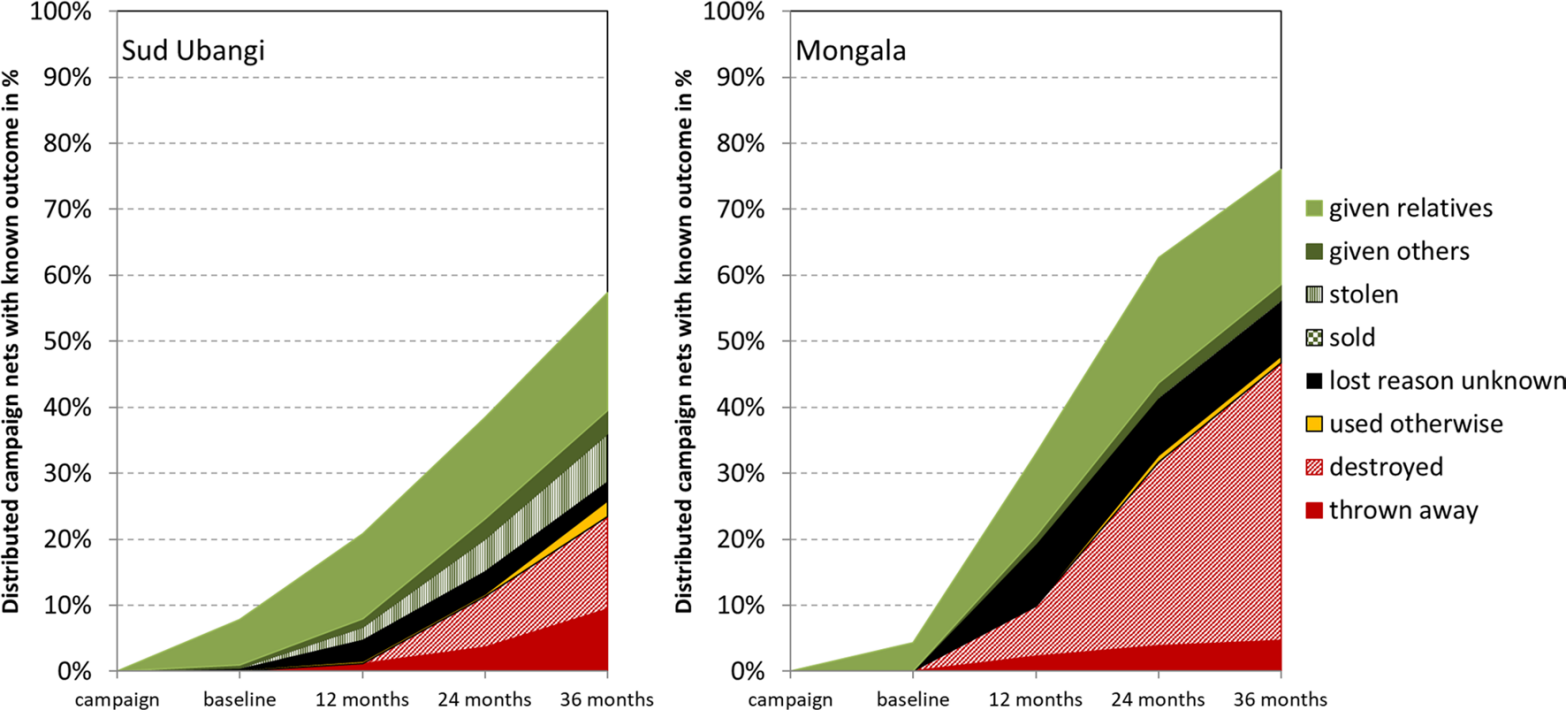
Attrition of cohort nets and their causes

The loss for any reason among cohort nets with a definite outcome, i.e. all-cause attrition, was 57% in Sud Ubangi at the end of the study and 76% in Mongala (p = 0.005). More importantly, attrition due to wear and tear (destroying, throwing away or use for other purposes) was 26% in Sud Ubangi and 48% in Mongala after 31 months (p = 0.0009). Details of the reasons for loss over time are shown in Fig. 3. The proportion of losses due to wear and tear among all losses was very small at baseline (0.2% Sud Ubangi and 0% Mongala) meaning that almost all losses were due to nets being given away to relatives or others. The proportion gradually increased and at the final survey 45% of all-cause attrition was due to wear and tear in Sud Ubangi and 63% in Mongala. In Sud Ubangi 21 nets received from the campaign were reported stolen (7%) but only one in Mongala. Reasons for loss among the discarded nets differed slightly between the sites (p = 0.018) with more discarded nets (36%) destroyed in Sud Ubangi or used for other purposes (10%) and the remainder (54%) thrown away. In contrast, only 11% of discarded nets in Mongala were destroyed and 2% used for other purposes while the bulk (87%) were thrown away. When calculated over all campaign nets with known outcome, the rate of alternative use was only 2% in Sud Ubangi and 1% in Mongala or 10 nets in total. All seven nets in Sud Ubangi were used as window or door curtains while two in Mongala were reportedly used for fishing and one to cover crops.

#### Integrity

The proportion of cohort nets still present in the surveyed households with any sign of damage initially increased rapidly but then levelled off as older nets were increasingly discarded, finally reaching 93% in Sud Ubangi and 78% in Mongala (Table 4). The proportion of nets with any damage was consistently higher in Sud Ubangi than in Mongala (p = 0.01) which is consistent with the lower hanging and use rates in Mongala. In contrast, the level of damage in nets with any holes, measured as median pHI, was significantly higher in Mongala at all time points (p < 0.0001). Nonetheless, the decline of the proportion of nets in serviceable condition was similar in both sites (p = 0.1) which was caused by the higher discard rate in Mongala.

**Table 4:** Integrity of campaign nets present in households

Variable	Baseline	12 months	24 months	36 months
	% (95% CI)	% (95% CI)	% (95% CI)	% (95% CI)
Sud Ubangi (DuraNet©)	N = 377	N = 269	N = 184	N = 122
Mean months since campaign	2.3	12.0	21.2	30.9
Net has any hole	9.3 (6.6–12.9)	61.3 (49.9–71.6)	85.9 (75.3–92.4)	93.4 (85.7–97.1)
Physical condition (pHI)				
Good (0–64)	98.9 (97.4–99.6)	72.9 (64.4–79.9)	37.0 (28.6–46.2)	23.0 (15.8–32.2)
Damaged (65–642)	1.1 (0.4–2.6)	17.5 (11.3–26.1)	29.9 (22.8–38.2)	36.1 (24.4–49.7)
Torn (> 642)	0 (-.-)	9.7 (6.1–14.9)	33.2 (24.2–43.5)	41.0 (31.0–51.7)
Serviceable (0–642)	100 (-.-)	90.3 (85.1–93.9)	66.9 (56.5–75.8)	59.0 (48.3–69.0)
Median pHI if any hole (IQR)	23 (2–25)	49 (8–265)	250.5 (54–2028)	438 (130–2113)
Has any repairs if any hole	0	4.2 (1.9–9.3)	8.3 (4.4–15.0)	15.8 (7.9–29.0)

Mongala (DawaPlus^®^ 2.0)	N = 377	N = 231	N = 106	N = 71
Mean months since campaign	2.1	12.1	12.3	30.6
Net has any hole	10.6 (5.1–20.9)	45.9 (33.0–59.3)	65.1 (46.2–80.2)	77.5 (60.0–88.8)
Physical condition (pHI)				
Good (0–64)	96.3 (91.2–98.4)	66.2 (54.5–76.3)	46.2 (31.5–61.6)	29.6 (14.8–50.4)
Damaged (65–642)	2.7 (1.3–5.3)	13.9 (9.1–20.5)	16.1 (11.2–22.4)	22.5 (12.1–38.1)
Torn (> 642)	1.1 (0.3–3.5)	19.9 (11.6–32.1)	37.7 (24.7–52.8)	47.9 (28.7–67.7)
Serviceable (0–642)	98.9 (96.5–99.7)	80.1 (67.9–88.4)	62.3 (47.2–75.3)	52.1 (32.3–71.3)
Median pHI if any hole (IQR)	48 (20–200)	466 (56–1362)	929 (246–2397)	1184 (219–3553)
Has any repairs if any hole	0	17.9 (11.4–27.1)	31.9 (18.0–49.9)	29.1 (15.3–48.3)

Complete or partial repairs of damaged cohort nets remained low even with increasing damage and were more common in Mongala with around 30% of damaged nets showing any repairs compared to a maximum of only 16% in Sud Ubangi (p = 0.002). The dominant method of repairing holes was stitching in Mongala with 88% of LLIN reported as repaired compared to 22% by knotting (some nets received both methods of repair), while in Sud Ubangi it was 55% and 59%, respectively. No patching was used in either site and repairs were exclusively done by family members or by relatives or friends. Households with damaged cohort nets that said they had never repaired holes were asked why they did not repair and among those that replied 27% said they did not know how or lacked materials for repair, 20% stated they had no time, and 19% felt it was not necessary or possible to repair with no difference between sites. Interestingly, a small amount of the campaign nets (8 in Sud Ubangi and 16 in Mongala, p = 0.003) were modified by their owners and this was mainly changing the shape from rectangular into a conical design.

#### Survival in serviceable condition

**Fig. 4:**
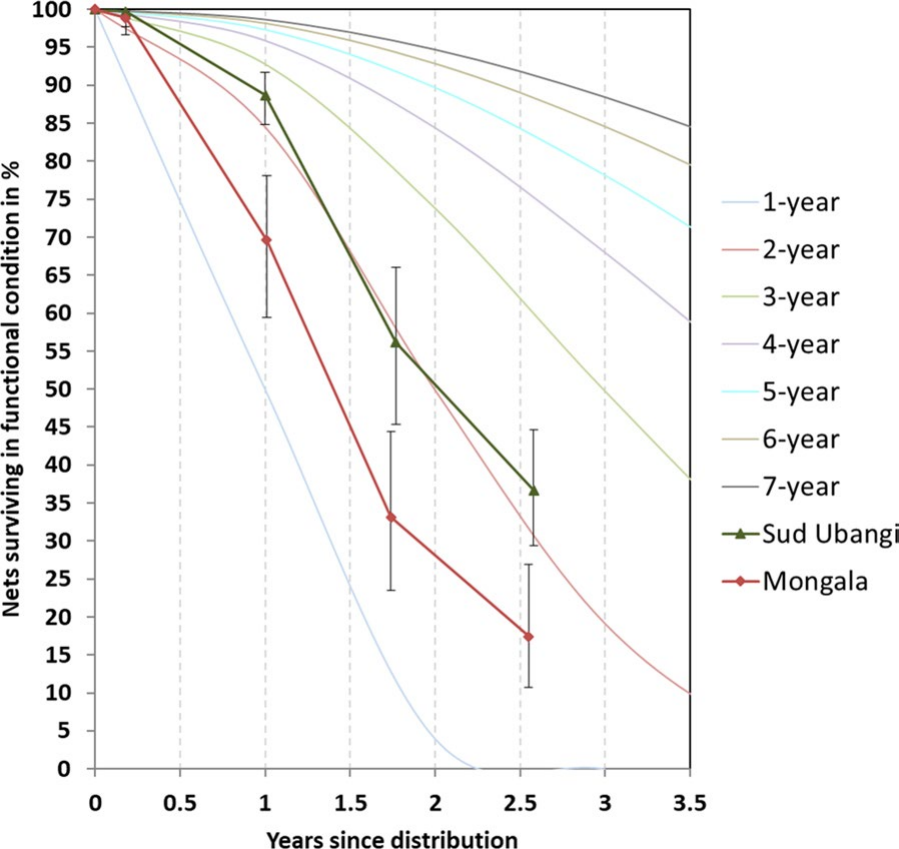
Survival of cohort nets in serviceable condition plotted against reference curves with defined median survival

The physical survival of LLINs in serviceable condition, i.e. combining attrition due to wear and tear and the integrity of the still existing LLINs, decreased rapidly over time (Table 5) and at the final survey, 31 months after distribution, was 37% in Sud Ubangi and only 17% in Mongala (p = 0.003). In order to facilitate comparisons with other durability data, the results were plotted against the hypothetical survival curves with defined median survival (Fig. 4). It can be seen that the survival estimates roughly follow the hypothetical curves and that the relationship between the two sites was the same throughout the time of follow-up. In addition to estimating median survival at each time point from the graph it was also calculated from the final two data points (see “methods”) and results are shown in Table 5. Calculated median survival was 1.6 years in Mongala (DawaPlus^®^ 2.0) and 2.2 years in Sud Ubangi (DuraNet©). Estimates obtained from the graph were very similar to those calculated at the final survey, but also show that in this setting earlier estimates from the graph at 12 and 24 months were comparable to the final estimate. Considering the confidence intervals around the median survival it can be said that at both sites performance of the tested LLIN was clearly below the three-year mark and in Mongala also below the 2-year mark.

**Table 5:** Estimated median survival in serviceable physical condition

Variable	12 months	24 months	36 months
Sud Ubangi (DuraNet©)			
% surviving in serviceable condition (95% CI)	88.7 (84.8–91.7)	56.2 (45.7–66.1)	36.7 (29.4–44.7)
Median survival in years			
Estimated from Fig. 4	2.4	1.9	2.2
Calculated from last two data points (95% CI)	-.-	-.-	2.2 (2.0–2.4)
Mongala (DawaPlus^®^ 2.0)			
% surviving in serviceable condition (95% CI)	69.6 (59.4–78.1)	33.2 (23.6–44.4)	17.4 (10.7–26.9)
Median survival in years			
Estimated from Fig. 4	1.5	1.4	1.6
Calculated from last two data points (95% CI)	-.-	-.-	1.6 (1.3–1.9)

**Fig. 5:**
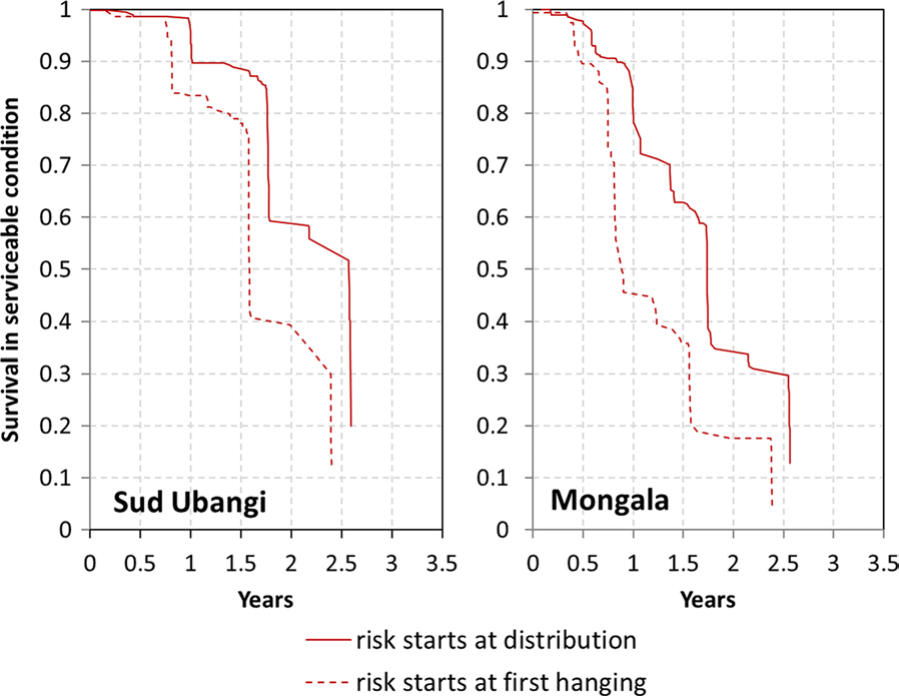
Kaplan-Meier survival functions of cohort nets comparing risk starting at distribution versus starting at first hanging

Kaplan–Meier survival curves comparing the intention to treat and per protocol analysis are presented in Fig. 5 and show a similar pattern of survival curves only shifted to the left by up to 1 years when risk of damage is considered to start only when the net is hung for the first time.

In both scenarios, the survival in Mongala was significantly lower than that in Sud Ubangi (p < 0.0001). Estimated median survival from the Kaplan–Meier function for Sud Ubangi was 1.0 years lower for the per protocol analysis compared to intention to treat (2.6 vs. 1.6) and for Mongala the difference was 0.8 years (1.7 vs. 0.9).

Determinants of physical survival from the Cox proportionate hazard models are presented in Table 6. In the multivariable model only considering household level factors the strong difference between sites was confirmed. In addition, having had a very positive net care attitude score at least once in any of the surveys was shown to have a significant protective effect with an adjusted Hazard Ratio (aHR) of 0.66. However, there was no dose–effect relationship as having a high attitude score at all observations did not have an additional effect. A protective effect was also seen in households headed by women (aHR 0.64) but here the evidence was not very strong (p = 0.14). No impact on survival was seen from exposure to SBCC messages, education of head of household, socio-economic tertile, discussing net care within the household, number of children, or household size.

**Table 6:** Determinants of physical durability (risk of failure to survive in serviceable condition) from Cox proportional hazard models; obs = observations

Variable	Adjusted hazard ratio (HR)	95% CI	P-value
At household level; N = 2176 obs/810 nets			
Site/Brand of LLIN			
Sud Ubangi (DuraNet)	1.00	1.85–3.56	< 0.0001
Mongala (DawaPlus 2.0)	2.57		
Net care attitude of household across surveys			
Never had a very positive score (> 1.0)	1.00	0.52–0.83	0.001
Had very positive score (> 1.0) at least once	0.66		
Gender of head of household			
Male	1.00	0.35–1.17	0.14
Female	0.64		
At net level (nets ever hung) N = 1603 obs/572 nets			
Site/Brand of LLIN			
Sud Ubangi (DuraNet)	1.00	1.82–3.95	< 0.0001
Mongala (DawaPlus 2.0)	2.68		
Net care attitude of household across surveys			
Never had a very positive score (> 1.0)	1.00	0.52–1.01	0.056
Had very positive score (> 1.0) at least once	0.72		
Users of net across all surveys			
Used by adults or together with children	1.00	1.30–2.93	0.003
Only used by children	1.95		
Type of sleeping place across all surveys			
Used over unfinished bedframe, foam mattress or	1.00	0.21–0.80	0.012
Reed mat Only been used over finished bedframe	0.41		

Adding net level variables, i.e. reducing the analysis to nets ever hung, reduced the impact of the positive net care attitude (aHR 0.72, p = 0.056), but the difference between sites remained significant. In addition, the use of nets exclusively by children increased the risk of failure to survive (aHR 1.95) while nets used exclusively over finished bedframes were protective (aHR 0.41). In this model, the effect of female headed household was no longer a significant determinant (p = 0.47) as it was closely associated with net users (in female headed households no children ever used the net on their own) as well as positive net care attitude. Net-related variables that did not have a significant impact on physical survival were folding or tying the net up during the day when hanging, storing food or cooking in the sleeping room, and drying nets over fences or bushes. Model diagnostics showed that the assumption of a proportionate hazard was met.

#### Insecticidal effectiveness

The target of sampling 30 campaign nets at each site and time point after baseline for bio-assay testing was achieved and results are shown in Table 7. While the DuraNet© LLIN brand maintained optimal performance throughout the three years of the study, the insecticidal effectiveness of the DawaPlus^®^ 2.0 LLIN brand only maintained high performance up to the 24-months data point and then dropped considerably with a median knock-down rate of 68% and median vector mortality of 26%. Only 10% of these samples showed optimal insecticidal performance, 47% minimal effectiveness and 53% failed even the minimal criteria. There was no evidence that the campaign nets collected at 12- and 24-months follow-up from neighbouring households differed from the cohort nets with respect net handling, use and washing patterns.

**Table 7:** Results from bio-assays using WHO cone test

Variable	12 months	24 months	36 months
Sud Ubangi (DuraNet©)	N = 30	N = 30	N = 30
Knockdown 60 min			
Mean (95% CI)	95.6% (93.5–97.6)	75.0% (67.3–82.7)	97.8% (96.5–99.1)
Median (IQR)	96.0% (92.0– 100)	74.0% (64.0–84.0)	99.0% (96.3–100)
Mortality 24 h			
Mean (95% CI)	89.6% (82.9–96.3)	92.2% (87.2–97.2)	84.8% (77.7–2.0)
Median (IQR)	98.5% (79.0–100)	94.0% (88.0–98.0)	89.1% (77.7–5.4)
Optimal effectiveness			
Estimate (95% CI)	83.3% (63.4–93.5)	86.7% (52.1–97.5)	100% (-.-)
Minimal effectiveness			
Estimate (95% CI)	100% (-.-)	100% (-.-)	100% (-.-)

Mongala (DawaPlus^®^ 2.0)	N = 30	N = 30	N = 30
Knockdown 60 min			
Mean (95% CI)	95.5% (92.7–98.2)	86.6% (81.1–92.0)	69.9% (61.0–8.8)
Median (IQR)	100% (92.0–100)	90.0% (78.0–94.0)	67.9% (60.5–9.5)
Mortality 24 h			
Mean (95% CI)	100% (-.-)	92.6% (88.8–96.4)	28.5% (21.1–5.8)
Median (IQR)	100% (-.-)	96.0% (88.0–100)	25.9% (10.7–1.7)
Optimal effectiveness			
Estimate (95% CI)	100% (-. -)	90.0% (72.3–96.9)	10.0% (2.9–28.9)
Minimal effectiveness			
Estimate (95% CI)	100% (-. -)	100% (-.-)	46.7% (30.9–3.1)

## Discussion

The objective of this cohort study was to compare the physical and insecticidal durability of two LLIN brands following their distribution through a mass campaign, one a 100-denier, multifilament polyester-based LLIN treated with deltamethrin (DawaPlus^®^ 2.0) and the other a 150-denier, monofilament polyethylene-based LLIN treated with alphacypermethrin (DuraNet©). To minimize the influence of net use environment and behavioural factors on LLIN durability two health zones very close to each other along the border between Sud Ubangi Province, which had received DuraNet©, and Mongala Province with DawaPlus^®^ 2.0, were selected as study sites. The proportion of LLIN physically surviving in serviceable condition was significantly lower in Mongala throughout the 31 months follow-up and at the final survey the proportion was 17% compared to 37% in Sud Ubangi. This corresponded to an estimated median survival time of 1.6–1.7 years in Mongala (depending on method used for estimation) and 2.2–2.6 yeas in Sud Ubangi. Although demographic, socio-economic and behavioural characteristics of the two populations were similar, there also were some important differences. More households in Sud Ubangi (33% vs. 1%) stated that they always cooked in the room where they also slept, but in the Cox proportionate hazard models this factor had no impact on physical survival. There were also more foam mattresses used as sleeping places in Mongala than Sud Ubangi, but again, there was no evidence from the multivariable models that this had an impact on physical survival in this setting. The major difference was a much higher level of positive attitude towards net care in Sud Ubangi where only 7% of households had never been found to have a very positive attitude score compared to 37% in Mongala even though exposure to SBC messages had been similar at both sites. A positive net care attitude had previously been found to have a positive impact of physical net durability in Nigeria [16] as well as Uganda [17] and this was also the case in this study. However, adjusting for the effects of the positive net care attitude in the multivariable model only reduced the hazard ratio comparing Sud Ubangi to Mongala by 0.2 from the bi-variable model (HR 2.9 vs. aHR 2.7) suggesting that the difference between sites was mainly due to the difference in LLIN brands. This allows the inference that in the given setting of poor, rural communities the DuraNet© performed significantly better than DawaPlus^®^ 2.0. No direct comparisons of physical durability of these two brands under field conditions has been published, but other comparisons between polyethylene and polyester LLIN brands exist. Using a comparable methodology Tan and colleagues [18] compared the 150-denier, polyethylene LLIN Olyset^®^ with the 100-denier, polyethylene LLIN PermaNet^®^ 2.0 in Luapula and Northern Provinces in Zambia and found a median survival of 2.0 years, but no differences between the brands. Similarly, Van Roey and colleagues [19] followed the 118-denier polyethylene LLIN Netprotect^®^ in Veal Veng District in Cambodia and compared it to the 100-denier, polyethylene PermaNet^®^. They found a similar physical survival in serviceable condition for both brands after three years of 58 and 61%, respectively, corresponding to a median survival of 3.2 and 3.4 years respectively. Another study did find a significant difference between LLIN brands, but only looked at the physical condition of surviving nets. Under the harsh conditions of camps for internally displaced people in Chad Allan et al. [20] found only 8% of 75-denier polyester LLIN (PermaNet^®^ and Interceptor^®^) were still in serviceable condition 14 months after distribution compared to 39% of the polyethylene LLIN Olyset^®^, i.e. a better performance the polyethylene LLIN similar to the findings of his study. This variation of findings in brand differences between locations suggests that textile characteristics of the LLIN other than yarn strength and material (e.g. knitting pattern) may play a significant role in the physical durability [21, 22]. Similarly, differences in performance between LLIN brands may only be apparent under poor environmental or net use conditions when the stress on the fabric is particularly high.

Estimated median survival of both brands monitored in this study remained well under the three-year mark, generally considered the average useful life of an LLIN. This is in keeping with the results of the unpublished durability study undertaken in DRC in 2015 [10]. A retrospective durability assessment of LLIN distributed through campaigns was done in eight provinces across DRC. Households with any children under five were randomly sampled and attrition and integrity of campaign nets were captured. Time since distribution varied between six and 44 months. The LLIN were mostly (89%) 100-denier PermaNet^®^ 2.0 with some Yorkool^®^ LLIN (same specifications as PermaNet^®^) and in Maniema Province also some Olyset^®^. Survival in serviceable condition was calculated and adjusted for recall bias of nets received from campaign [15] assuming households received one net for every two people.

Median survival of campaign nets was estimated from the hypothetical loss curves also used in this study. In Equateur Province, neighbouring Sud Ubangi and Mongala Provinces, estimated median survival 30 months after distribution of PermaNet^®^ 2.0 was 1.7 years, very similar to what was found for the polyester LLIN DawaPlus^®^ 2.0 in this study. In central DRC (Kasaï Occidentale) and the northeast (Maniema and Orientale) median survival was even lower at 1.3–1.4 years. Only in the western DRC around the capital Kinshasa were higher survival rates found, around 2.0 years in Kinshasa and Bas-Kongo Provinces, and only in Bandundu Province the median survival estimate reached the three-year mark with 3.1 years. Low estimates of physical median survival of LLIN have also been reported from other locations and for other LLIN brands. In Zambia Olyset^®^ and PermaNet 2.0^®^ had an estimated median survival of 2.0 years [23], Olyset^®^ in Benin 1.5–2.0 years [18] and PermaNet 2.0^®^ in Ethiopia only 1.0 years [24]. On the other hand, median survival times well above three years have also been reported. In Nigeria, a retrospective durability study of the DawaPlus^®^ 2.0 LLIN found median survival varying between 3.0 and 4.7 years in three sites [15] and these results were recently confirmed in a prospective study also in Nigeria with median survival of DawaPlus^®^ 2.0 ranging from 3.2 to 5.3 years (Obi et al. pers. commun.). In Kenya, median survival of Olyset^®^ was measured as 4.0–4.5 years [25] and in Cambodia a median survival for PermaNet^®^ 2.0 was 3.4 years [19]. This range of physical durability results for the same of similar brands of LLIN is much wider than any differences observed between LLIN brands discussed above and suggests that differences in net use and care environment are more important for the durability outcome than differences in netting material. This also implies that while physical durability in DRC currently appears to be low, it would be expected to improve with socio-economic development and improved net care behaviours.

Two months after distribution many campaign nets in this study were not yet hanging and between 43% (Sud Ubangi) and 71% (Mongala) were found still in their packages. Hanging rates thereafter increased rapidly and at the final survey 82% in Sud Ubangi and 55% in Mongala had been found hanging at least once. Such delays in first hanging and use have been described also in other settings and are thought to be primarily a function of availability of other nets already in use and the household’s decision whether to switch immediately or wait until the old nets are worn [26]. Indeed, over 80% of non-cohort nets in both sites were hung at baseline and at later surveys hanging and use rates of cohort and non-cohort nets were similar. Mongala, which had the lower rate of ever hung cohort nets, also had a higher proportion of non-cohort nets among all nets owned and when all nets in the household were considered, hanging rates at the final survey were 83% in Sud Ubangi and 75% in Mongala. This shows that the lower hanging rate in Mongala was mostly due to the higher availability and utilization of non-cohort nets. Another factor that contributed to a lower hanging of nets in Mongala at the end of the study was the finding that with increasing damage the nets were hung less and damage levels in Mongala were higher than in Sud Ubangi. This phenomenon of damaged nets being used less has previously also been described in Ethiopia [27, 28].

Insecticidal effectiveness of the DuraNet©, measured by the WHO cone bio-assay, remained high throughout the follow-up and exceeded 80% optimal performance at the 31 months assessment. Similar results have also been reported for DuraNet© from northern Tanzania where these nets still had 94% mortality in bio-assays after 20 washes [29]. In contrast, bio-assay results for DawaPlus^®^ 2.0 showed 90% optimal performance at 24 months but then deteriorated and at the final survey only 10% showed optimal effectiveness and 53% failed to reach even minimal effectiveness. Unfortunately, no published studies of bio-assays or chemical residue of DawaPlus^®^ 2.0 over time under field conditions exist beyond the studies submitted for the WHO evaluation of the product [12]. However, in a recent prospective durability study in Nigeria this LLIN brand showed 97% optimal effectiveness in two sites in Nigeria after 36 months (Obi et al. submitted). It is not clear whether this was a gradual decline of available insecticide in and on the netting or a dramatic loss between 24 and 36 months surveys as no chemical residue testing was done in this study and the high bio-assay results at 24 months could also have been achieved at relatively low levels of deltamethrin [30]. From the findings of this study no obvious reason for the poor insecticidal durability of the DawaPlus^®^ 2.0 LLIN in this setting is apparent. Washing rates in Mongala were moderate with on average four washes per year, i.e. estimated 12 washes in 3 years and there was no excessive use of detergent. One factor that could have contributed to a faster decline in insecticide is the observation that in Mongala over 80% of the cohort nets were always dried outside and potentially exposed to sunlight. This has been shown to reduce insecticide content by up to 5%-points in a study in Kenya [31]. However, another study has demonstrated that when bio-assays are used as outcomes repeated exposure to sun only reduces vector mortality if the exposure is 3 days after each wash, but not if the drying period is only 3 h [32]. This makes it unlikely that the outside drying in Mongala alone can explain the observed decline in insecticidal effectiveness. However, taking into account the poor physical durability of DawaPlus^®^ 2.0 with only 17% surviving in serviceable condition after 31 months and the surviving nets in poor condition and used less and less before being discarded, it is very unlikely that the low insecticide levels in this instance had a significant public health impact.

### Limitations

Some of the durability risk factors such as net care and repair attitude as well as some of the outcomes such as reason for net loss were based on the answers of the household members interviewed and, therefore, are prone to recall or social desirability biases. With the prospective design there is also the potential for the Hawthorne effect, whereby being asked about net care and handling four times over the course of 3 years may have contributed to changes in behaviour. The standard durability monitoring approach tries to minimize this by conducting only four surveys vs every 6 months as had been done in some of the earlier studies. Furthermore, while the sample of the campaign net cohort was representative for the selected health zones within each province, the health zone selection was purposive and some caution is required when generalizing the findings to the province or even DRC as a whole.

## Conclusions

After 31 months of follow-up among rural populations, the 150-denier polyethylene LLIN DuraNet© showed a significantly better median physical survival compared to the 100-denier polyester LLIN DawaPlus^®^ 2.0, but both remained well under the three-year expected median survival. The difference could be attributed mostly to the differences in brand itself and to some extent to positive net care behaviour, use by adults only and use exclusively over a finished bedframe. In harsh environments in DRC such as these, it appears to be preferable to distribute a 150-denier polyethylene LLIN, such as the DuraNet© or similar brands. Intensified SBCC around net care behaviours would also be able to improve physical durability of the LLIN distributed. Insecticidal performance was optimal for DuraNet©, but for the DawaPlus^®^ 2.0 optimal performance lasted only up to 24 months and failed at 36 months. However, at this time most of the cohort nets had been already lost or were no longer serviceable.

## Supplementary information

**Additional file 1.** Hypothetical loss functions with defined median survival. Presents detailed graph, formula and parameterization of hypothetical loss functions.

**Additional file 2.** Household characteristics. Contains table with demographic and socio-economic characteristics of sampled households.

## Data Availability

The datasets used and/or analysed during the current study are available from the corresponding author on reasonable request.

## Abbreviations

ANC: Ante-natal care
EPI: Expanded programme for immunization
HR: Hazard ratio
IPC: Inter-personal communication
ITN: Insecticide-treated net
LLIN: Long-lasting insecticidal net
NMCP: National Malaria Control Programme
pHI: Proportionate hole index
SBCC: Social and behaviour change communication
