# Supplementary material for: Comparing the durability of the long-lasting insecticidal nets DawaPlus® 2.0 and DuraNet© in northwest Democratic Republic of Congo
Source: Malar J. 2020 May 24;19:189. doi: 10.1186/s12936-020-03262-0 (PMC7247235; doi:10.1186/s12936-020-03262-0)
Supplement: Supplementary file 2 — Additional file 2. Household characteristics. Contains table with demographic and socio-economic characteristics of sampled households. [file 12936_2020_3262_MOESM2_ESM.pdf]

Additional file 2

Household characteristics

Table: Household characteristics (based on households that were seen at baseline and endline surveys)

Variable	Sud Ubangi	Mongala	P-value
	% (95% CI)*	% (95% CI)*	for comparison between sites
**Demographic**			
Mean number of de-jure members	7.3 (6.7-7.9)	6.2 (5.8-6.6)	0.005
Mean age of head of household in years	44.5 (40.5-48.2)	43.3 (37.8-49.0)	0.75
Proportion of female headed households	11.3 (4.6-25.1)	7.4 (4.2-12.7)	0.40
Proportion of under-fives	21.8 (19.2-24.7)	16.5 (13.1-20.7)	0.037
Education of male heads of household			
Non-literate	29.2 (19.5-41.1)	15.4 (8.1-27.3)	
Primary	16.7 (11.6-23.3)	25.3 (18.2-34.0)	0.10
Secondary	54.2 (41.5-66.3)	59.3 (45.7-71.6)	
**House characteristics**			
Improved roof materials	14.9 (4.5-39.7)	21.0 (8.3-43.7)	0.61
Improved wall materials	5.4 (0.8-28.4)	18.5 (5.9-45.2)	0.20
Improved floor materials	1.2 (0.2- 4.8)	13.0 (3.8-35.6)	0.003
Cooking fuel kerosene or gas	0.0 (-.-)	0.0 (-.-)	n.a.
**Water and sanitation**			
Access to safe water	0.0 (-.-)	18.5 (5.5-47.3)	0.070
Access to any latrine	98.2 (95.2-99.4)	99.4 (95.5-99.9)	0.32
Improved latrine or flush toilet	0.0 (-.-)	0.0 (-.-)	n.a.
**Household assets**			
Any transport	47.2 (34.6-59.8)	55.6 (42.3-68.1)	0.35
Type of transport			
Bicycle	44.1 (32.3-56.5)	48.2 (34.8-61.7)	0.65
Motorcycle	9.5 (3.9-21.6)	16.7 (9.6-27.4)	0.25
Car	0.0 (-.-)	0.0 (-.-)	n.a.
Owns farm land	70.8 (49.7-85.7)	64.8 (49.6-77.5)	0.61
Owns livestock	91.7 (83.6-96.0)	79.6 (73.9-84.4)	0.015
Type of livestock			
Chicken	79.3 (69.6-86.5)	69.9 (61.2-77.5)	0.12
Goats	27.4 (19.5-37.1)	17.9 (11.5-26.7)	0.11
Cows	1.2 (0.1- 9.2)	0.0 (-.-)	0.35
Household items owned			
Radio	42.3 (29.9-55.7)	53.7 (42.6-64.4)	0.19
Television	0.6 ((0.1- 4.9)	7.4 (3.2-15.7)	0.007
Refrigerator	0.0 (-.-)	0.0 (-.-)	n.a.
Fan	0.0 (-.-)	0.0 (-.-)	n.a.
Iron	0.0 (-.-)	0.0 (-.-)	n.a.
Any mobile phone	7.7 (3.4-16.8)	22.2 (12.6-36.2)	0.03
Smartphone or computer	1.2 (0.3- 4.5)	0.0 (-.-)	0.14
